# Increased expression of stefin B in the nucleus of T98G astrocytoma cells delays caspase activation

**DOI:** 10.3389/fnmol.2012.00093

**Published:** 2012-09-14

**Authors:** Tao Sun, Vito Turk, Boris Turk, Nataša Kopitar-Jerala

**Affiliations:** ^1^Department of Biochemistry, Molecular and Structural Biology, Jožef Stefan InstituteLjubljana, Slovenia; ^2^Liaoning Cancer Hospital and InstituteShenyang, Liaoning, PR China

**Keywords:** cathepsin, cell death, cystatin, EPM1, histone

## Abstract

Stefin B (cystatin B) is an endogenous inhibitor of cysteine proteinases localized in the nucleus and the cytosol. Loss-of-function mutations in the stefin B gene (CSTB) gene were reported in patients with Unverricht-Lundborg disease (EPM1). Our previous results showed that thymocytes isolated from stefin B-deficient mice are more sensitive to apoptosis induced by the protein kinase C (PKC) inhibitor staurosporin (STS) than the wild-type control cells. We have also shown that the increased expression of stefin B in the nucleus of T98G astrocytoma cells delayed cell cycle progression through the S phase. In the present study we examined if the nuclear or cytosolic functions of stefin B are responsible for the accelerated induction of apoptosis observed in the cells from stefin B-deficient mice. We have shown that the overexpression of stefin B in the nucleus, but not in the cytosol of astrocytoma T98G cells, delayed caspase-3 and -7 activation. Pretreatment of cells with the pan-caspase inhibitor z-Val-Ala-Asp(OMe)-fluoromethylketone completely inhibited caspase activation, while treatment with the inhibitor of calpains- and papain-like cathepsins (2S,3S)-trans-epoxysuccinyl-leucylamido-3-methyl-butane ethyl ester did not prevent caspase activation. We concluded that the delay of caspase activation in T98G cells overexpressing stefin B in the nucleus is independent of cathepsin inhibition.

## Introduction

The cystatins are endogenous inhibitors of the papain-like cysteine cathepsins and are localized mostly in the cytosol or secreted from the cells (Turk et al., 2002; Abrahamson et al., 2003; Kopitar-Jerala, 2006). Cysteine cathepsins are involved in protein degradation (Turk et al., 2000) and in the development and function of the immune system (Hsing and Rudensky, 2005).

Loss-of-function mutations in the stefin B gene are found in the patients with Unverricht-Lundborg disease (EPM1), but the physiological function of stefin B in the molecular pathogenesis of the disease is not known (Lalioti et al., 1997; Pennacchio et al., 1996). Stefin B-deficient mice display a phenotype similar to the human disease, with progressive ataxia and myoclonic seizures (Pennacchio et al., 1998). Although, stefin B has long been known to inhibit papain-like cathepsins *in vitro* by tight and reversible binding, the physiological function of stefin B in the molecular pathogenesis of the disease remains unknown. Houseweart et al. showed that simultaneous deletion of cathepsin B and stefin B greatly reduced the neuronal apoptosis in mice, but did not rescue the ataxia and seizure phenotypes, and concluded that there are other factors besides cathepsin B that are involved in the disease pathology (Houseweart et al., 2003). Cysteine cathepsins have been reported to contribute to apoptosis induced by various stimuli in several cell types (Foghsgaard et al., 2001; Stoka et al., 2001; Turk et al., 2012). It was shown that after their release from the lysosomes, the cathepsins cleave Bid thereby activating it and allowing it to induce the mitochondrial release of cytochrome *c* and subsequent apoptosis (Repnik et al., 2012). However, it was demonstrated that in stefin B-deficient mice Bid signaling is not essential for the apoptosis in cerebellum (Houseweart et al., 2003).

We showed that thymocytes isolated from stefin B-deficient mice were more sensitive to staurosporin (STS)- induced apoptosis than the wild-type thymocytes and the mechanism of apoptosis in this particular case is independent of cathepsins and dependent only on caspases (Kopitar-Jerala et al., 2005). In our previous study we have identified an interaction between stefin B and the nucleosomes, specifically with the histones H2A.Z, H2B, and H3. In the synchronized T98G cells, stefin B co-immunoprecipitated with histone H3, predominantly in the G1 phase of the cell cycle (Čeru et al., 2010). The increased expression of stefin B in the nucleus delayed cell cycle progression in T98G cells, while stefin B-deficient mouse embryonic fibroblasts entered the S phase earlier than the wild-type mouse embryonic fibroblasts. The delay in cell cycle progression was associated with the inhibition of cathepsin L in the nucleus, as judged from the decreased cleavage of the CUX1 transcription factor.

The aim of our present study was to evaluate how does the overexpression of stefin B into the nucleus influence STS-induced apoptosis. Stefin B contains a putative 14-3-3 binding site and we investigated whether the binding of stefin B to 14-3-3 proteins was responsible for the delay of caspase activation. The latter are intracellular, acidic dimeric molecules that play a role in signal transduction pathways and apoptosis (Gardino and Yaffe, 2011). In general, 14-3-3 proteins play a role in promoting survival and repressing apoptosis (Morrison, 2009).

We conclude that the mechanism, by which stefin B in the nucleus delays apoptosis, is independent of cysteine cathepsins and 14-3-3 proteins, but we cannot exclude the possibility that the interaction with histones, or specific histone variants are important for the process.

## Materials and methods

### Cell culture

T98G human glioblastoma cell line, ATCC CRL-1690, was from the American Type Culture Collection (Manassas, VA). Cells were cultured as described previously (Čeru et al., 2010) T98G cells were transfected with pEF/Myc/Nuc/stefin B named NB or empty pEF/Myc/Nuc vector alone named NO, using Lipofectamine 2000 (Invitrogen), according to the manufacturer's instructions. Positive clones overexpressing stefin B in the nucleus were obtained after selection with Geneticin (G418) (Invitrogen), as described before (Čeru et al., 2010).

### Bright field microscopy

T98G cells were grown in DMEM, 10% FCS, and 2 mM l-glutamine in 6-well culture plates at 5 × 10^6^ cells/well. The cell-permeable inhibitors E-64d (Peptide Institute, Osaka, Japan), or z-VAD-fmk (Bachem AG, Switzerland) were added in adequate concentrations in dimethylsulfoxide (DMSO) 1 h prior to induction of apoptosis. The corresponding volume of DMSO was added to the control cultures. To induce apoptosis, cells were treated 500 nM STS for 5 h. Live and death cells were determined with an Olympus IX81 microscope (Olympus, Japan) and by MTS (3-(4,5- dimethylthiazol-2-yl)-5-(3-carboxymethoxyphenyl)-2-(4-sulfophenyl)-2H-tetrazolium) viability assay.

### MTS reduction assay

Cell viability was assayed by MTS viability assay (Promega, Fitchburg, WI), essentialy as described previously (Anderluh et al., 2005). The T98G cells were plated on to 96-well plates at a density of 10^4^ cells/well in 100 μL DMEM medium. The cell-permeable inhibitors E-64d (Peptide Institute, Osaka, Japan), or z-VAD-fmk (Bachem AG, Switzerland) were added in adequate concentrations in DMSO 1 h prior to induction of apoptosis. The corresponding volume of DMSO was added to the control cultures. Cell death was induced wih 500 mM STS. Twenty microliters of MTS reagent was then added to each well. The plate was incubated for 2–3 h at 37°C in a 5% (v/v) CO_2_ humidified environment. Four independent experints were performed in triplicates. The absorbance of formazan was measured at 490 nm.

### Measurement of DEVD-ase activity

Fifty micrograms samples of protein from control and STS-treated cells, in the presence or absence of inhibitors, were used to determine caspase activity by measuring proteolytic cleavage of the fluorogenic substrate Ac-DEVD-trifluoromethylcoumarin (Bachem), as described (Kopitar-Jerala et al., 2005).

### Preparation of cell lysates and western blots

Cell lysates were prepared as described (Kopitar-Jerala et al., 2005). The cell lysates were transferred to fresh test tubes and, if not used immediately, stored at −80°C. Total protein concentration was determined using the Bradford assay (Bio-Rad). Western blots were performed as described (Kopitar-Jerala et al., 2005). Equal amounts of protein were loaded and resolved in 15 or 12.5% SDS-PAGE gels and electrotransferred to nitrocellulose membranes. Proteins were visualized with ECL (Amersham) according to the manufacturer's instructions. All immunoblots were probed with actin to confirm equal protein loading.

### Statistical analysis

Data are presented as mean ± S.D. Statistical differences among groups were evaluated by the Student *t*-test. Differences were considered significant for *P* < 0.005.

## Results

### STS-induced apoptosis T98G cells is caspase-dependent and cathepsin-independent

**Figure 1:**
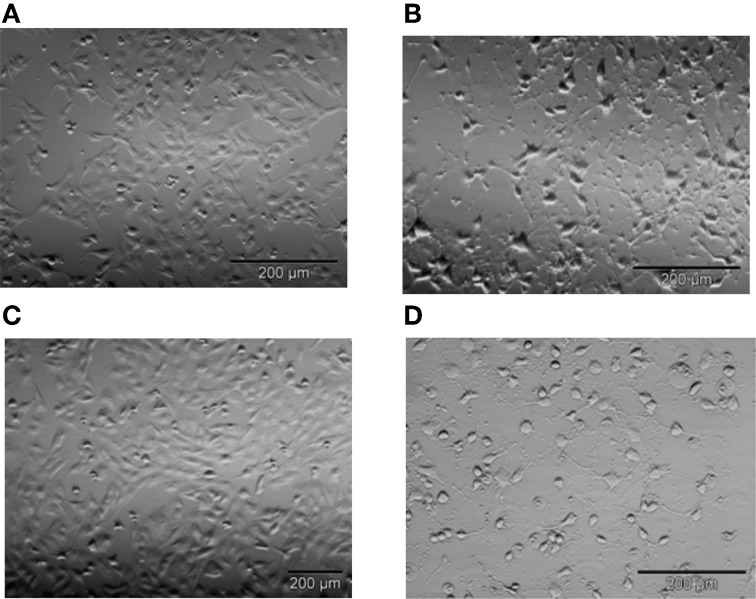
**Representative images of T98G cells after 5 h of incubation with 500 nM STS.** Following treatment with 500 nM STS cells shrunk, rounded, and detached from the surface as seen by light microscopy **(A)** untreated cells **(B)** cells treated with 500 nM STS. The majority of these morphological changes were prevented by Z-VAD-fmk **(C)**, but not by E-64d **(D)**. Bar 200 μM.

We first confirmed that STS-induced apoptosis in T98G cells follows the same mechanism as STS-induced apoptosis in thymocytes (Kopitar-Jerala et al., 2005). In our previous experiments we determined the dose response curve of STS on T98G cells, and used 500 nM STS. Treatment of T98G astrocytoma cells with 500 nM STS for 9 h resulted in detachment of cells from the culture dishes and major morphological changes, such as considerable cell shrinkage and rounding (Figure 1). All of the morphological changes were substantially prevented by pretreatment of cells with the pan-caspase inhibitor Z-VAD-fmk (25 μM), but not with the general inhibitor of cysteine cathepsins E-64d (25 μM) indicating that STS-induced apotosis in T98G cells is caspase-dependent and cathepsin-independent (Figure 1).

**Figure 2:**
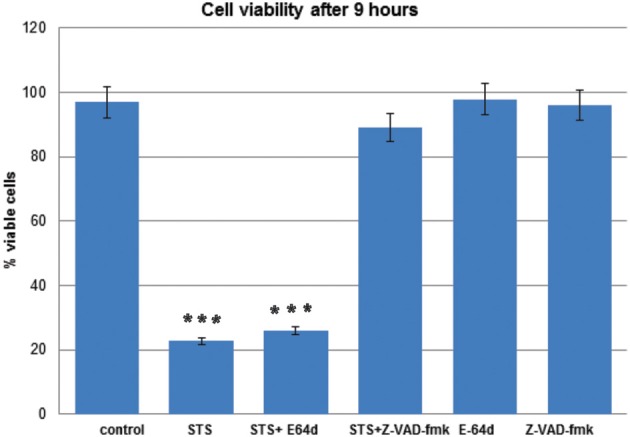
**T98G cell viability upon STS treatment.** MTS assays were performed to measure the T98G cell viability after STS treatment. Data are expressed by the mean of percent cell viability compared to control after exposure for 9 h ± standard deviation. The cell-permeable inhibitors E-64d or z-VAD-fmk were added in adequate concentrations in DMSO 1 h prior to induction of apoptosis. The corresponding volume of DMSO was added to the control cells. Cell death was induced wih 500 mM STS. The data are representative of three independent experiments. Statistical significance was analyzed by the Student's *t*-test. A *P*-value ≤ 0.001 (^***^) was considered significant.

Decrease in cell viability after STS treatment and pretreatment with the inhibitors was determined quantitatively using the MTS assay, as described (Anderluh et al., 2005). T98G cells were treated with STS in the presence or the absence of the pan-caspase inhibitor z-VAD-fmk or E-64d. In T98G cells z-VAD-fmk inhibited cell death, while E-64d did not have any effect on cell survival (Figure 2).

### Expression of stefin B in the nucleus delayed caspase activation

**Figure 3:**
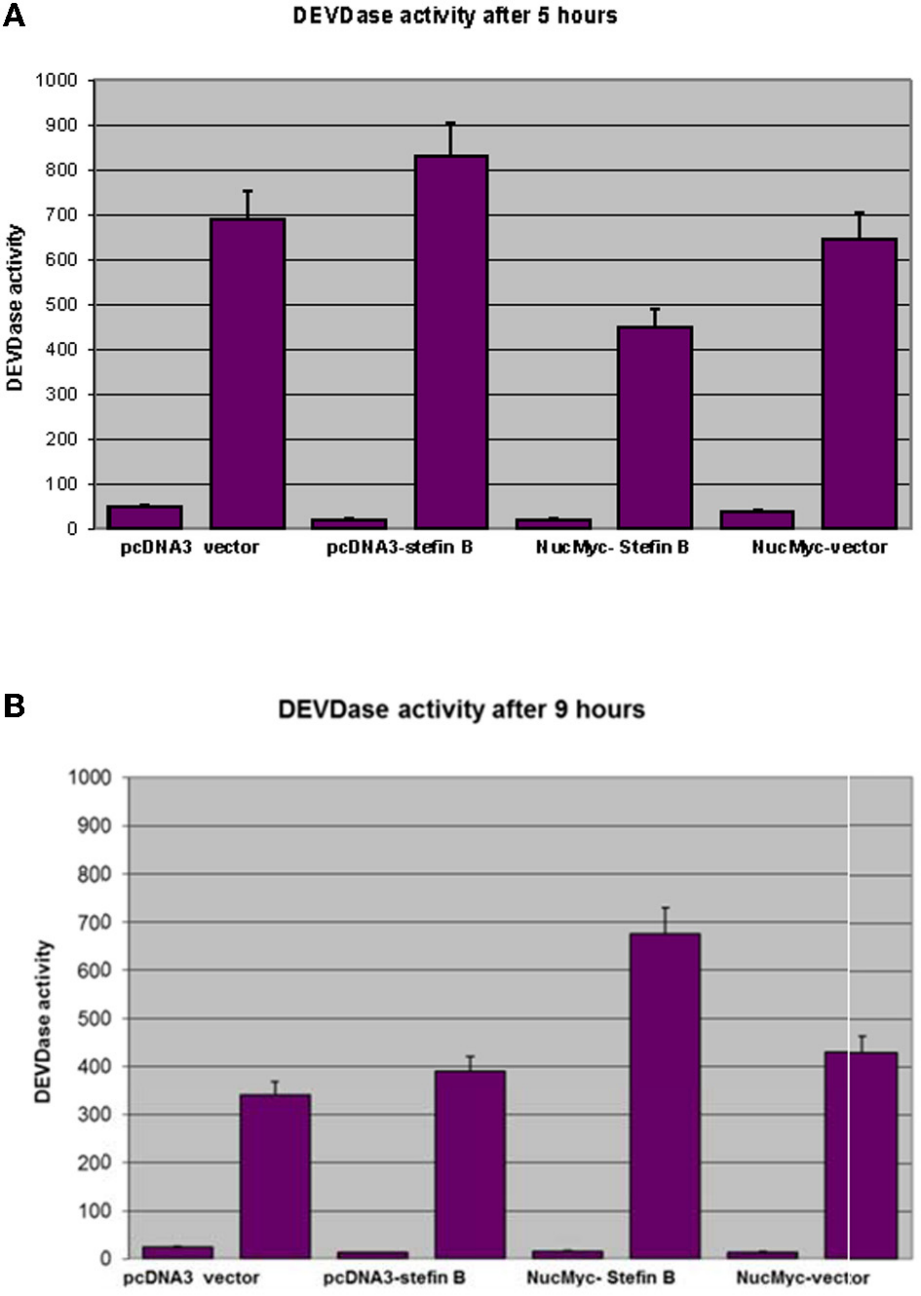
**DEVD-ase activities in the cell lysates.** T98G cells transfected with an empty vector (pcDNA3 vector), T98G cells overexpressing stefin B in the cytosol (pcDNA3-stefin B) and in the nucleus (NucMyc-stefin B), together with a control empty vector pEF/myc/nuc (NucMyc-Vector) were treated with 500 nM STS. DEVD-ase activities were determined after 5 h **(A)** and 9 h **(B)** of incubation, as described in Materials and Methods. Results are means ± S.D. of at least three independent experiments performed in triplicate.

**Figure 4:**
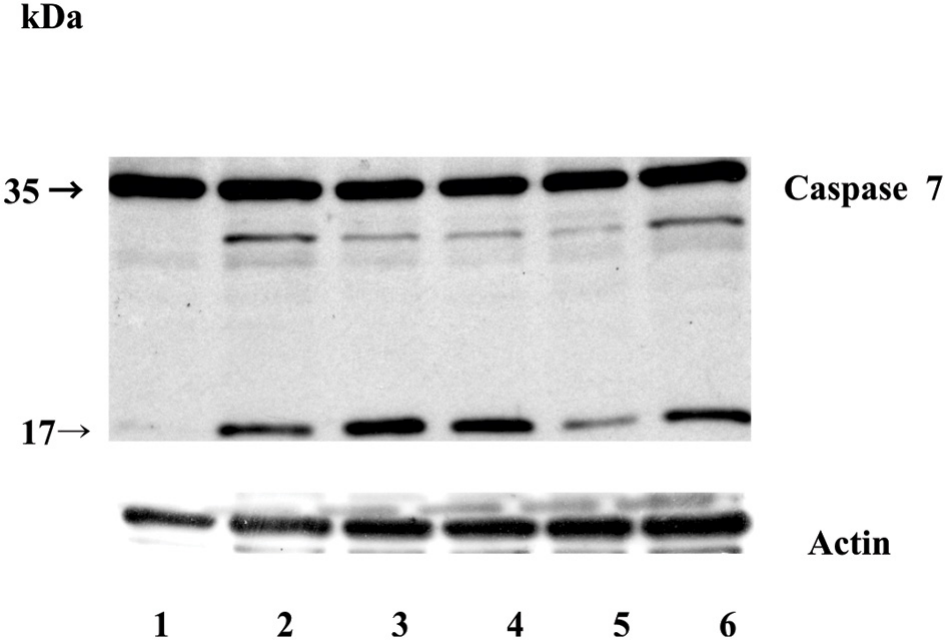
**Caspase-7 activation during STS-induced apoptosis in T98G cells.** Equal amounts of proteins (70 μg) were loaded and separated on 12,5% SDS-PAGE, followed by Western blotting with anti-caspase-7 monoclonal antibody F5/1 (Gregorc et al.). Membranes were stripped and re-developed with anti-actin Ab. Track 1: control (untreated cells); 2: T98G cells; track 3: T98G with an empty vector pcDNA3, track 4: T98G cells overexpressing stefin B into the cytosol; track 5: T98G cells overexpressing stefin B into the nucleus (NB); track 6: control cells transfected with an empty pEF/myc/nuc vector (NO). The data represent the mean of triplicate data points ± S.E. and are representative of three independent experiments. The data are representative of three independent experiments.

Next, we examined whether overexpression of stefin B in the nucleus influences STS-induced apoptosis. T98G cells were transfected with a pEF/Myc/Nuc vector expressing stefin B pEF/Myc/Nuc-stefin B (NB), or an empty vector alone (NO) as described (Čeru et al., 2010). We followed the time course of apoptosis with regard to caspase activation and cell viability testing. Examination of the time-dependent activation of DEVD-ase activity induced by STS revealed that DEVD-ase activity began to increase approximately 5 h after STS treatment and reached a maximum after 9 h. Five hours after addition of STS a significantly faster caspase activation was observed in the control T98G cells and T98G cells with increased expression of stefin B into cytosol, but not NB cells overexpressing stefin B into nucleus (Figure 3A). Nine hours after the addition of STS, the DEVD-ase activity was increased in NB cells, while in the other control cell lines it was already lower than at the 5 h time point (Figure 3B). The same was observed both in T98G transiently transfected cells, and in the stable cell lines obtained after G418 selection. Apoptotic cell death was additionally confirmed with Western blotting of caspase-7 activation (Figure 4). Since apoptosis is cathepsin-independent, we reasoned that the non-inhibitory functions of stefin B are responsible for the effect we observed.

### Increased expression of stefin B in the nucleus or cytosol did not prevent PARP cleavage by caspases

**Figure 5:**
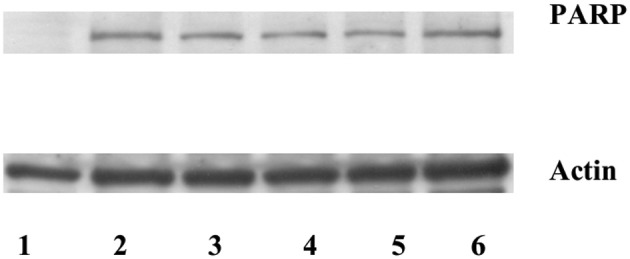
**Caspase substrate PARP cleavage was detected after STS treatment in T98G cells and T98G cells transfected with stefin B.** An equal amount of proteins (70 μg) was loaded and separated on 12,5% SDS-PAGE, followed by Western blotting with the anti-PARP antibody. Membranes were stripped and re-developed with anti-actin Ab. Track 1: control (untreated cells); track 2: T98G cells; track 3: T98G with an empty vector pcDNA3, track 4: T98G cells overexpressing stefin B into cytosol; track 5: T98G cells overexpressing stefin B into nucleus; track 6: control cells transfected with an empty pEF/myc/nuc vector.

Poly(ADP-ribose) polymerase (PARP) is a well defined substrate of caspases during the apoptosis. Since the increased expression of stefin B in the nucleus, but not in the cytosol, delayed caspase activation, we examined the cleavage of a nuclear caspase substrate Poly(ADP-ribose) polymerase (PARP). Using specific antibodies we detected the specific PARP fragment (85 kDa) generated by the caspases in the T98G cell lysates prepared 5 h after the induction of apotosis (Figure 5). However, we could not confirm any significant differences between PARP cleavage in the control cells and in the cells overexpressing stefin B in the nucleus or in the cytosol (Figure 5).

### Stefin B does not interact with 14.3.3 proteins

**Figure 6:**
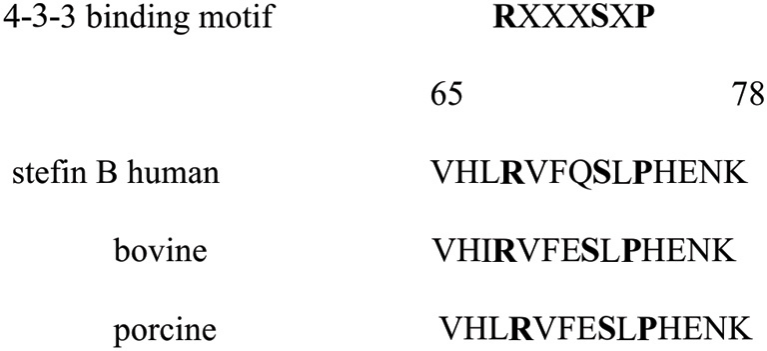
**Stefin B species variants contain putative 14-3-3 binding site**.

The stefin B amino acid sequence contains the conventional binding motif that is present in most 14-3-3 binding proteins, and is also conserved in several stefin B species variants (Figure 6). We investigated if the interaction of stefin B with 14-3-3 proteins is important for the delay of apoptosis observed in the T98G cells with increased nuclear expression of stefin B. However, the interaction of stefin B with 14-3-3 proteins could not be confirmed by co-immuno precipitations neither in the cytosolic nor in the nuclear extracts (data not shown), suggesting that the delay in caspase activation and apoptosis in s not due to the interactions of stefin B with 14-3-3 proteins.

## Discussion

The results of our study provide a new insight into the regulation of cell death by stefin B, a nuclear and cytosolic inhibitor of cysteine cathepsins. It has been shown that the increased expression of stefin B in the nucleus, but not in the cytosol, only delays caspase activation and apotosis, but did not prevent cell death.

STS activates the intrinsic apoptotic pathway, it induces mitochondrial outer membrane permeabilization, which is followed by the release of cytochrome *c* and the downstream activation of caspases (Duan et al., 2003). Cells from mice lacking both Bax and Bak are completely resistant to STS, as well as several other apoptotic stimuli that act through disruption of mitochondrial function (Wei et al., 2001). Moreover, recent studies demonstrated that the cathepsins, when released from the lysosomes degrade not only Bid, but also the antiapoptotic Bcl-2 family members Bcl-2, Bcl-xL, and Mcl-1, thereby also triggering the mitochondrial apoptotic pathway (Droga-Mazovec et al., 2008). Several reports showed that lysosomal cysteine cathepsins, in particular cathepsin B, participate also in the extrinsic apototic parhway induced by the Tumor necrosis factor (TNF)-related apoptosis-inducing ligand (TRAIL) (Nagaraj et al., 2006; Werneburg et al., 2007). TRAIL induces apoptosis by binding to two death receptors TRAIL-R1 and TRAIL-R2. This leads to the recruitment of the adaptor protein, Fas-associated death domain (FADD), which in turn recruits the initiator caspase, caspase-8, which is activated by auto-proteolytic cleavage, leading to the activation of downstream effector caspases and cell death (Pop et al., 2007). In addition, stefin B was also suggested to contribute to the resistance of melanoma cells to TRAIL-induced apoptosis, however, the inhibition of cathepsins L and B was found not to be important in this process, and stefin B was suggested participate in the process by preventing the proteasomal degradation of FLIP_L_, by an yet undefined mechanism (Yang et al., 2010). Leist and Jäättelä suggested that the cathepsins can mediate also caspase-independent apoptosis (Leist and Jäättelä, 2001), but in the present study we demonstrated that STS-induced apoptosis in T98G cell was completely independent of cysteine cathepsins and dependent only on the caspases. Our findings are in agreement with the proposal that the cathepsins predominantly trigger the caspase-dependent cell death (Repnik et al., 2012).

Another potential mechanism, by which stefin B could contribute to the delayed STS-induced apoptosis are reactive oxygen species (ROS). It was reported that STS-induced apoptosis leads also to the generation of ROS (Ricci et al., 2004). Moveover, it was shown that stefin B deficiency sensitizes cerebellar granule neurons to oxidative stress-induced cell death (Lehtinen et al., 2009). It could be therefore expected that overexpression of stefin B in the cytosol would protect the cells from the ROS-induced cell death. However, we did not detect any significant difference in caspase activity in cells with increased expression of stefin B in cytosol and in the control cells. We can conclude that at least in the system used, ROS did not play a major role in caspase activation.

Another potential link between apoptosis and stefin B present also the proteins from 14-3-3 family. STS inhibits several protein kinases, however, it has the highest affinity for the protein kinase C (PKC) isoenzymes (Tamaoki et al., 1986). Recent reports have shown that 14-3-3 proteins function as a PKC regulators (Saurin et al., 2008). While 14-3-3 proteins often interact with the phosphoserine or phosphothreonine residues in their ligands, they can also interact with some ligands, including human telomerase (Seimiya et al., 2000) and the amyloid beta-protein precursor intracellular domain fragment (Sumioka et al., 2005), in a phosphorylation-independent manner. Although stefin B contains a putative 14-3-3 binding site, the interaction of stefin B with 14-3-3 proteins was not detected in the cytosol or in the nucleus.

However, we cannot exclude the possibility that the interaction of stefin B with histones, or specifically with modified histones, could be responsible for the delay in caspase activation. Histones can be covalently modified by several post-translational modifications including acetylation, methylation, phosphorylation, and ubiquitination (Kouzarides, 2007). These modifications occur mainly in the amino-terminal tail of the histones, but can also occur in the globular domain. The “histone code” hypothesis proposes that specific histone modifications regulate gene expression by either changing the chromatin structure into an “activated” or “repressed” transcriptional state, or by acting as a docking site for transcriptional regulators that associate with chromatin (Jenuwein and Allis, 2001; Loyola and Almouzni, 2007). We have reported that stefin B is associated with H2A.Z and H3K4Me3 histone variant (Čeru et al., 2010). H2A.Z variant was found in the promoter regions associated with regulatory elements (Barski et al., 2007). It has been further suggested that H2A.Z deposition and H3K4me3 modification may facilitate nucleosome eviction or repositioning in promoter regions of the human genes (Schones et al., 2008). H3K4me3 histone modification has been shown to facilitate pre-mRNA maturation (Sims et al., 2007), implying a possible role for stefin B in this process.

In conclusion, we have shown that stefin B overexpression in the nucleus delayed not only cell cycle progression, as reported before, but also caspase activation. Whereas the main reason for the delayed cell cycle progression is probably the decreased CUX1 cleavage, the mechanism by which nuclear stefin B delays caspase activation is not clear yet. Stefin B was thus shown not to interfere with ROS signaling nor to interact with the 14-3-3 proteins, which regulate the PKC function. One of the possibilities remain the stefin B interaction with the histones. However, additional studies will be needed to reveal the mechanism by which nuclear stefin B delays caspase activation.

### Conflict of interest statement

The authors declare that the research was conducted in the absence of any commercial or financial relationships that could be construed as a potential conflict of interest.

## Abbreviations

Ac-DEVD-AMC: acetyl-Asp-Glu-Val-Asp-AMC
AFC: 7-amino-4-trifluoromethyl coumarin
AMC: 7-amino-4-methyl coumarin
DMSO: dimethylsulfoxide
MTS: (3-(4,5- dimethylthiazol-2-yl)-5-(3-carboxymethoxyphenyl)-2-(4-sulfophenyl)-2H-tetrazolium)
ROS: reactive oxygene species
STS: staurosporin
z-VAD-fmk: benzyloxycarbonyl-Val-Ala-Asp-fmk
fmk: fluoromethyl ketone
E-64d: (2*S*,3*S*)-*trans*-epoxysuccinyl.
